# TRIPS flexibilities help change policy and practice to increase access to medicines: evidence from 2001 to 2024

**DOI:** 10.1136/bmjgh-2025-021481

**Published:** 2026-01-28

**Authors:** Montgomery Dunn, Ellen ’t Hoen, Pascale Boulet, Kaitlin Mara, Katrina Perehudoff

**Affiliations:** 1 University of Cambridge, Cambridge, UK; 2 Medicines Law & Policy, Amsterdam, Netherlands; 3 Global Health, University Medical Centre, Groningen, Netherlands; 4 Law Centre for Health and Life, University of Amsterdam, Amsterdam, Netherlands

**Keywords:** Health Services Accessibility, Global Health, Health policy, Review

## Abstract

**Introduction:**

Millions of people lack access to safe and effective pharmaceuticals because they are unaffordable or unavailable, particularly in ‘developing’ and ‘least-developed’ countries (DCs, LDCs), and increasingly in high-income countries (HICs). Management of intellectual property (IP) related to new medicines has a significant impact on access to safe, affordable and effective medicines. The Agreement on Trade-Related Aspects of Intellectual Property Rights (TRIPS) provides the international legal framework for IP protection and mandates 20-year patents in all technological fields, including pharmaceuticals. TRIPS contains flexibilities, such as compulsory licensing (CL) and transition provisions for LDCs, which governments can use to facilitate access to health technologies. The use of these flexibilities is underreported in the literature, and a thorough analysis has not been undertaken since the COVID-19 pandemic.

**Methods:**

A scoping review of three medical and legal databases and temporal analysis of all known instances of use or potential use of CLs and the LDC pharmaceutical transition measure between 2001-2024.

**Results:**

61% of the 149 CL instances were executed. The relative rates of CL use between countries have shifted: HICs represent over half of CL instances in the last decade. CLs are increasingly considered for chronic, non-communicable and rare diseases. The threat of CL use continues to provide impetus for price negotiations, voluntary licences or other measures to improve access. Almost all eligible countries have invoked the right to use the LDC transition measure.

**Conclusions:**

TRIPS flexibilities have been used to facilitate access to medicines (including vaccines) over the quarter-century since the adoption of the World Trade Organization’s Doha Declaration on TRIPS and Public Health. The flexibilities play a vital role in ensuring that new medicines are affordable and are likely to continue to be in a future where geopolitical forces have drastically altered the financing structures of medicines provision in DCs and LDCs.

**WHAT IS ALREADY KNOWN ON THIS TOPIC**

**WHAT THIS STUDY ADDS**

- This study of 2001–2024 presents the most comprehensive review of uses or potential uses of compulsory licensing and the LDC transition measure since the adoption of the Doha Declaration on TRIPS and Public Health, including previously unreported instances and validation of all instances.
- TRIPS flexibilities remain a routine tool for WTO Members to improve access to medicines, with 199 reported instances between 2001 and 2024 (mostly compulsory licensing (n=149) and the LDC pharmaceutical transition measure (n=46)).
- Compulsory licensing activity in high-income countries has increased over time, driven by high-priced treatments for cancer, rare diseases and heightened interest during the COVID-19 pandemic.
- No countries have publicly invoked their right to use the LDC pharmaceutical transition measure since 2009, likely due to the absence of notification requirements and possibly due to the expanded use of voluntary licences.

**HOW THIS STUDY MIGHT AFFECT RESEARCH, PRACTICE OR POLICY**

- This paper should inform the WTO’s 30-year review of the TRIPS Agreement, as proposed by Colombia and currently under consideration.
- High-income country governments should continue to widen the policy space for their use of TRIPS flexibilities to increase access (such as by opting back into the Article 31bis mechanism) and must refrain from implementing TRIPS-plus provisions in trade agreements.
- Compulsory licensing provisions need to include measures to waive additional intellectual property rights (eg, data exclusivity) if necessary to give effect to these licences.
- Future research should examine the direct and indirect impacts of instances of executed and non-executed flexibilities, particularly those where the applicant is civil society.

## Introduction

 Millions of people lack access to safe and effective medicines^1^,^3^ often because they are unaffordable or unavailable, particularly in ‘developing’ and ‘least-developed’ countries (DCs and LDCs). Overcoming this challenge is to an important degree determined by intellectual property restrictions that shape whether healthcare systems and individuals can access safe, effective and affordable (generic) medicines.^4 5^

Since the World Trade Organization’s (WTO) establishment in 1995 and the adoption of the WTO Agreement on Trade Related Aspects of Intellectual Property Rights (TRIPS Agreement), almost all WTO Members have been obliged to provide a minimum 20-year patent protection in all technological fields, including medicines. This protection gives patent holders a temporary monopoly, delaying market competition and the entry of lower-cost generic products. Health crises such as the HIV and COVID-19 pandemics have shown that market monopolies on medical products cost lives.^4^

The 2001 Doha Declaration on the TRIPS Agreement and Public Health^6 7^ recognised both the importance of intellectual property for the development of new medicines, and the tension between IP protection and public health needs. The Doha Declaration reaffirmed the right of countries to use TRIPS flexibilities^8^ (the areas of policy space in which WTO Members can determine the way in which TRIPS provisions are implemented, accommodating national interests^9^). Between 2001 and 2016, TRIPS flexibilities, particularly for HIV treatments, were used widely by countries to secure an affordable supply.^10^

Since 2016, the global determinants of access to medicines have shifted: rising medicines prices,^11^ the shift in global disease burden towards more chronic conditions and non-communicable diseases (NCDs)^12^ and the trend towards isolationist politics and aid cuts.^13 14^ The onset of the COVID-19 pandemic and the response and recovery periods may have significantly altered the use of TRIPS flexibilities globally. For example, the global shortage of COVID-19 vaccines and therapies further legitimised states’ use of TRIPS flexibilities to secure a supply of medicinal products.^15^ Since the pandemic struck, some wealthy countries, particularly in the European Union (EU), appear to be shifting their stance on the use of TRIPS flexibilities, as seen in recent legislative and policy changes.^16 17^ Meanwhile, a growing number of states are experiencing conflict (eg, Ukraine) and may seek to use TRIPS flexibilities to safeguard their national interests and access to healthcare through generic competition, imports and self-sufficiency in production.^18^

In 2023, the 24th WHO Expert Committee for the Selection of Essential Medicines made it explicit that it would not include several effective medicines for the treatment of cancer due to their high prices. In making this statement, it referenced the potential role of voluntary licensing in reducing prices.^19^ In early 2025, the USA announced it would halt all funding for PEPFAR (US President’s Emergency Plan For AIDS Relief) and significantly reduce funding for all overseas aid,^20^ though it has since announced a limited waiver.^21^ In 2024 and 2025, many other high-income countries (HICs) announced large cuts to their foreign aid budgets, many of which support medicines research, testing and/or provision to DCs and LDCs.^22 23^

These global shifts in disease burden, political pressures and global health financing structures warrant a thorough examination of the use of TRIPS flexibilities by states worldwide, as their importance seems likely to increase.

Meanwhile, in certain disease areas, the need for TRIPS flexibilities may be decreasing on account of voluntary licensing (VL) (ie, a patent holder grants a third party the right to make use of its own rights under certain conditions). The Medicines Patent Pool (MPP) was founded in 2010 to ‘pool’ VLs to allow generic manufacturers to produce affordable medicines for supply to DCs (and LDCs). Initially, the MPP’s mandate covered HIV medicines only,^24^ before expanding to include hepatitis C and tuberculosis in 2015^25^ and all essential patented medicines in 2018.^26^ In the last 15 years, the MPP has negotiated over 30 VLs for essential medicines.^27^ For the first time in 2022, the MPP’s portfolio included an NCD medicine: nilotinib, indicated for Chronic Myeloid Leukaemia.^28^ The broadened mandate of the MPP may have had an impact on the use of TRIPS flexibilities that further warrants investigation.

This paper aims to document the use of TRIPS flexibilities, focusing on compulsory licensing (CL) and the LDC pharmaceutical transition measure to increase access to affordable medicines between 2001 and 2024 and to examine how these uses have evolved over this time. It also includes illustrative examples of the use of other TRIPS flexibilities.

## Methods

A scoping review was conducted to identify instances of the potential or actual use of TRIPS flexibilities applied to the procurement and supply of medicines since 2001, updating and expanding on ‘t Hoen *et al*^10^ by including new data from 2017 onwards and previously unreported cases from 2001 to 2016. The relevant measures for facilitating access to medicines are defined in box 1.

Box 1 **Types of TRIPS flexibilities for facilitating access**

**Compulsory licensing (CL) (including public non-commercial use/government use): TRIPS Article 31**

The authorisation granted by a government authority to use a patented invention by a third party during the patent term without the consent of the patent holder, usually against the payment of a reasonable remuneration and after attempts to obtain a VL have failed. The latter is not a requirement where the CL is issued for public non-commercial use or in case of a national emergency or other circumstances of extreme urgency. Public non-commercial use licences are a type of CL issued by the government for its own use or for the use of a third party. As per TRIPS Article 44.2, remedies available against use of a patented invention without the consent of the patent holder may be limited to payment of remuneration only.

**CL for export: TRIPS Article 31(bis)**

A special form of CL designed to lift the export restrictions of Article 31(f) which states that products produced under a CL shall be predominantly for the supply of the domestic market of the granting nation. This restriction hampers the effective use of CL by countries that depend on importation of products produced under a CL to give effect to a CL issued for their own territories. Article 31bis contains a mechanism to grant a CL for export to eligible countries. Many HICs opted out of the provision as importers. Article 31bis was the first, and so far only, amendment to the TRIPS Agreement in 2017. It was previously referred to as the Paragraph 6 system, for its location in the Doha Declaration.

**Least developed countries pharmaceutical transition measure: TRIPS Article 66.1, Doha Declaration Paragraph 7**

Least developed country (LDC) Members of the WTO can postpone the granting and/or enforcement of pharmaceutical patents until at least 2033. No payment of compensation is required. LDCs lose this right if they graduate from LDC status before this deadline.^57^

Parallel Importation*: TRIPS Article 6, Doha Declaration Paragraph 5(d)

The importation and resale of a patented product from another country without the consent of the patent holder. Parallel importation is based on the principle of international exhaustion which means that once the patent holder has put a product on the market anywhere in the world, he/she has exhausted the right to control it. Countries are free to determine whether they use an international, national or regional (eg, EU) exhaustion regime.

Exceptions to Patent Rights* - TRIPS Article 30

An example of a patent exception often found in patent law is the exception for research and experimentation. This exception shields researchers from patent infringement suits when they carry out experiments with a patented invention. Article 30 is also the basis for the regulatory (Bolar) exception. An Article 30 exception is automatic and does not involve having to seek the consent of the patent holder.

*Parallel importation and the use of the patent right exception may be available for procurement purposes in countries that provide for them in their national law. They do not require notification. As such, tracking their use over time is difficult. While the LDC transition measure also does not require notification, many LDCs elected to make known their use of this measure for reasons highlighted in the discussion. Therefore, this study only attempts to comprehensively cover the use of CLs and the LDC transition measure.HICs, high-income countries; TRIPS, Trade-Related Aspects of Intellectual Property Rights; VL, voluntary licensing.

‘Medicines’ are understood as medicinal products defined as ‘A substance or combination of substances that is intended to treat, prevent or diagnose a disease, or to restore, correct or modify physiological functions by exerting a pharmacological, immunological or metabolic action’,^29^ which are used to treat humans.

A scoping review was selected because evidence in this field is fragmented across disciplines.

### Literature search

The literature search was conducted to identify previously unreported cases (ie, those not included in the Medicines Law & Policy (ML&P) TRIPS Flexibilities Database^30^). According to the protocol (see online supplemental annex), designed in line with the Preferred Reporting Items for Systematic reviews and Meta-Analyses (Systematic review) PRISMA-Sr guidelines,^31^ three databases were searched (Ovid Medline, Ovid Embase, LexisNexis) in May 2024 (see online supplemental annex S1). All publications (1 January 2024–1 May 2024) were retrieved. In addition, publications were collected from the reference lists of included articles (citation chaining).

### Inclusion criteria

Inclusion criteria were papers concerning (1) at least one medicine^29^ for human use and (2) at least one instance when authorities have invoked, planned to invoke or have been formally asked to invoke a TRIPS flexibility of relevance (see above) for public interest reasons. There were no exclusion criteria.

For the LDC transition measure, this means that the instances presented likely do not represent every time that the flexibility has been used—but rather where there is a known notification of their use (likely the first time).

### Selection of studies

The search results were combined, and duplicates detected by Rayyan reference management software^32^ were removed.

Papers were screened in two stages. First, title and abstract screening was independently performed by two blinded reviewers. Then, full texts were screened for inclusion by two blinded reviewers. Discrepancies were discussed with a third author.

### Data extraction and appraisal

Data were extracted (where available) on the country, year, type of flexibility, product(s), therapeutic indication, applicant, whether the flexibility was executed and what the outcome was. Where this information was not available, the authors searched references and publicly available information.

Where publicly accessible information could not be found to verify a particular instance, the authors spoke to officials or experts with direct knowledge and/or involvement in the instance. Authors discussed cases with no evidence to theorise the likely misunderstanding and recorded these discrepancies.

Possible instances were sorted into ‘false’ or ‘true’ instances if they referred to a specific patented product(s), and could be verified as one of the following events: (1) a government announcement of the intent to invoke a TRIPS flexibility; (2) a request or application by a third party to invoke a TRIPS flexibility and (3) the actual use of a TRIPS flexibility.

### Patient and public involvement

Not applicable to this study.

### Analysis

The analysis includes the instances already documented in the ML&P Database and the previously unreported cases between 2001 and 2024.

Verified instances were categorised by type of flexibility; type of applicant; countries where the instances were considered; therapeutic indication reported in official documents (if unavailable, then by their primary indication listed in the Electronic Medicines Compendium^33^); status of execution (ie, executed, non-executed and pending if there was no clear concluding outcome and the case was less than 5 years old). Executed status was awarded if the flexibility was implemented (eg, a CL was issued) but does not necessarily mean the product was procured through this method.

Countries were classified by WTO country classification, which was regarded as mutually exclusive—as LDCs (n=37),^34^ DCs (n=90) (DCs)^35^ and high-income countries (n=39) (HICs). The WTO maintains a list of LDCs. Countries self-identify as ‘developing’ within the framework of the WTO.^36^ This study classified all other countries as HICs. TRIPS flexibilities have also occasionally been used by non-Members of the WTO and WTO Observers,^37^ which are presented separately in the data.

**Figure 1:**
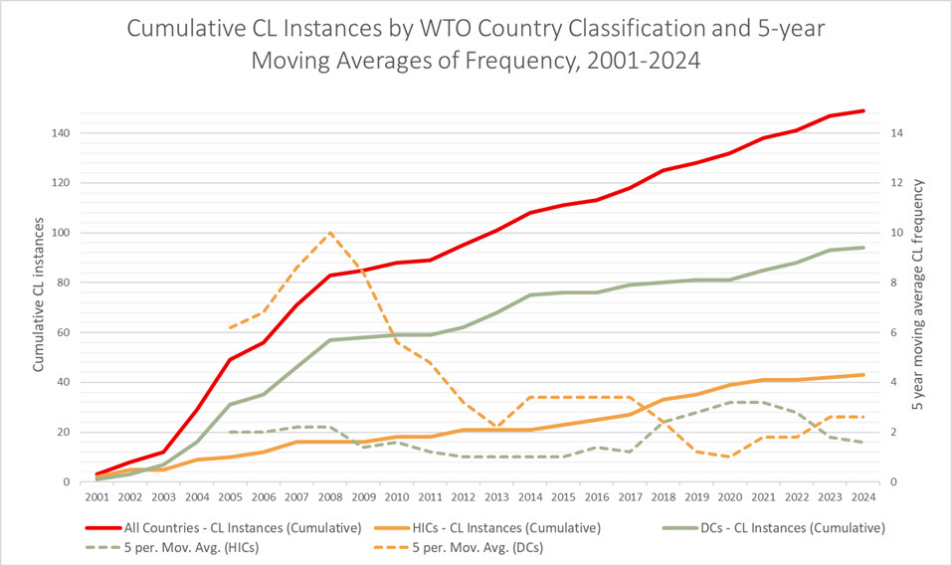
Cumulative instances of CLs between 2001 and 2024 and the 5-year moving averages of frequency. CL, compulsory licensing; DCs, developing countries; HICs, high-income countries.

A descriptive temporal analysis reports data aggregated by the categories above. Trends over time were analysed and related to relevant contextual factors. Case studies of particular medicines and flexibilities are used to illustrate relevant points. A 5-year moving average is used in figure 1 to smooth fluctuations in the yearly rate and allow for a medium-term trend analysis. A proportion z-test is used to compare the rates of CL use between HICs and DCs as the populations are independent with binary outcomes (executed or not executed).

See the Glossary (online supplemental file 2) for further definitions. Raw data are accessible in the ML&P Database.^30^

## Results

The search and citation chaining yielded a total of 622 results, which, after removing duplicates, resulted in 594 papers that underwent abstract and title screening. Of these, 379 papers were excluded, leaving 215 papers for full-text review. Of these, 78 papers were excluded because they did not identify a specific TRIPS Flexibility instance (n=65) or they could not be accessed using multiple institutional logins (n=13). 137 papers referred directly to a specific (not yet verified) instance of one or more TRIPS flexibilities as defined above for a human medicine. See online supplemental annex S2,S3 for the PRISMA flow chart and a full list of included papers.

From the 137 included papers, 39 potentially new instances (ie, previously unreported in the ML&P Database) were identified. During verification, 23 were found not to be true instances because the information available was not a formal request, application for or use of a TRIPS flexibility (n=21), or there was no information available (n=2).

Combining the results of the search with the existing data in the ML&P Database, between 2001 and 2024, 199 instances of TRIPS flexibilities were reported worldwide. Of these, most were compulsory licences (n=149) and use of the LDC pharmaceutical transition measure (n=46), which can be comprehensively monitored while (parallel import, n=1 and research exception, n=3) cannot (see table 1). 61% of the compulsory licences were executed (see Section 3: outcomes of recorded instances of TRIPS flexibilities).

**Table 1:** Instances of compulsory licences and use of the LDC transition measure and the percentages of executed CLs, 2001–2024

Flexibilities instances: 2001–2024		
Type of measure	Instances identified *n (%)*	Executed CLs *n (%)*
Total	195 (100)	
CL: Total	149 (76.4)	91 (61.1)
CL: Local supply	144 (73.8)	90 (60.4)
CL: Article 31bis	5 (2.6)	1 (20.0)
LDC transition measure	46 (23.6)	

CL, compulsory licensing; LDC, least-developed country.

### Section 1: the use of different TRIPS flexibilities over time

In the period 2001–2024, there have been more instances of compulsory licence use, announcement or request than instances of use of the LDC transition measure (see table 1). Most CL instances (n=149) have been for local supply (n=144), with a minority utilising the TRIPS Article 31bis (n=5) mechanism for export (see online supplemental annex S4 for details on these cases).

New instances of LDC measures were used between 2003 and 2009, after which their use stagnated.

#### Use of compulsory licences (Articles 31 and 31bis)

The combined yearly rate of CL instances was highest between 2003 and 2008, and since then it has continued at a relatively stable rate of approximately four CL instances per year. However, while the combined yearly rate has remained largely constant, the countries responsible have shifted.

Figure 1 demonstrates the shift in the type of countries utilising CLs to facilitate access to medicines.

Although still less common than in DCs, instances of CLs in HICs significantly increased from 15% of total CL use in the decade between 2005 and 2014 to 54% between 2015 and 2024 (p<0.01, proportion z-test).

The 5-year moving average of CL instances in DCs peaked in 2008 and since has remained approximately stable (approx. 2 instances/year). The 5-year moving average was higher in HICs than DCs in the period 2018–2022. HICs, which make up less than a fifth of the world’s population,^38^ have represented over half of CL instances in the last decade.

Of the five recorded instances of the Article 31bis mechanism, three concern export from Canada (2004 imatinib; 2006 oseltamivir; 2007 combination therapy of Lamivudine-Azidothymidine-Nevirapine) and two instances are paired representing the importing and exporting countries (Canada-Bolivia 2021 COVID-19 vaccine).^30^ Only one instance (2007) was executed for export to Rwanda, which relied on the LDC transition measure for medicines importation.^30^ The other four instances were not executed due to non-response and administrative delays adding the product to Schedule 1, as required by Canadian law (see online supplemental annex S4).^30^

#### Use of the LDC pharmaceutical transition measure (‘Paragraph 7 mechanism’)

Between 2004 and 2009, 46 instances of invocation of the LDC transition measure (see box 2) were reported in 27 of the 33 countries classified as LDC WTO Members and five countries that were either not WTO Members or not LDCs. Of all instances, 34 relate specifically to HIV/AIDS medicines, whereas the other 12 instances apply to all medicines.

Box 2 **TRIPS special provisions for least developed countries (LDCs)^58^**

Under Article 66.1 of the TRIPS Agreement, LDC Members of the WTO benefit from a transition period that allows them to delay fully implementing the TRIPS Agreement in their national law. The deadline for the transition has been extended three times and currently is 1 July 2034. Separately, as per the Doha Declaration on TRIPS and Public Health, LDCs may postpone the granting and/or enforcement of pharmaceutical patents until at least 1 January 2033 or until the country ceases to be an LDC. This separate pharmaceutical-related measure is particularly useful for LDCs that are already TRIPS compliant and/or grant pharmaceutical patents because they are not obliged to enforce such patents.

TRIPS, Trade-Related Aspects of Intellectual Property Rights.

### Section 2: therapeutic indications for instances of compulsory licensing

**Figure 2:**
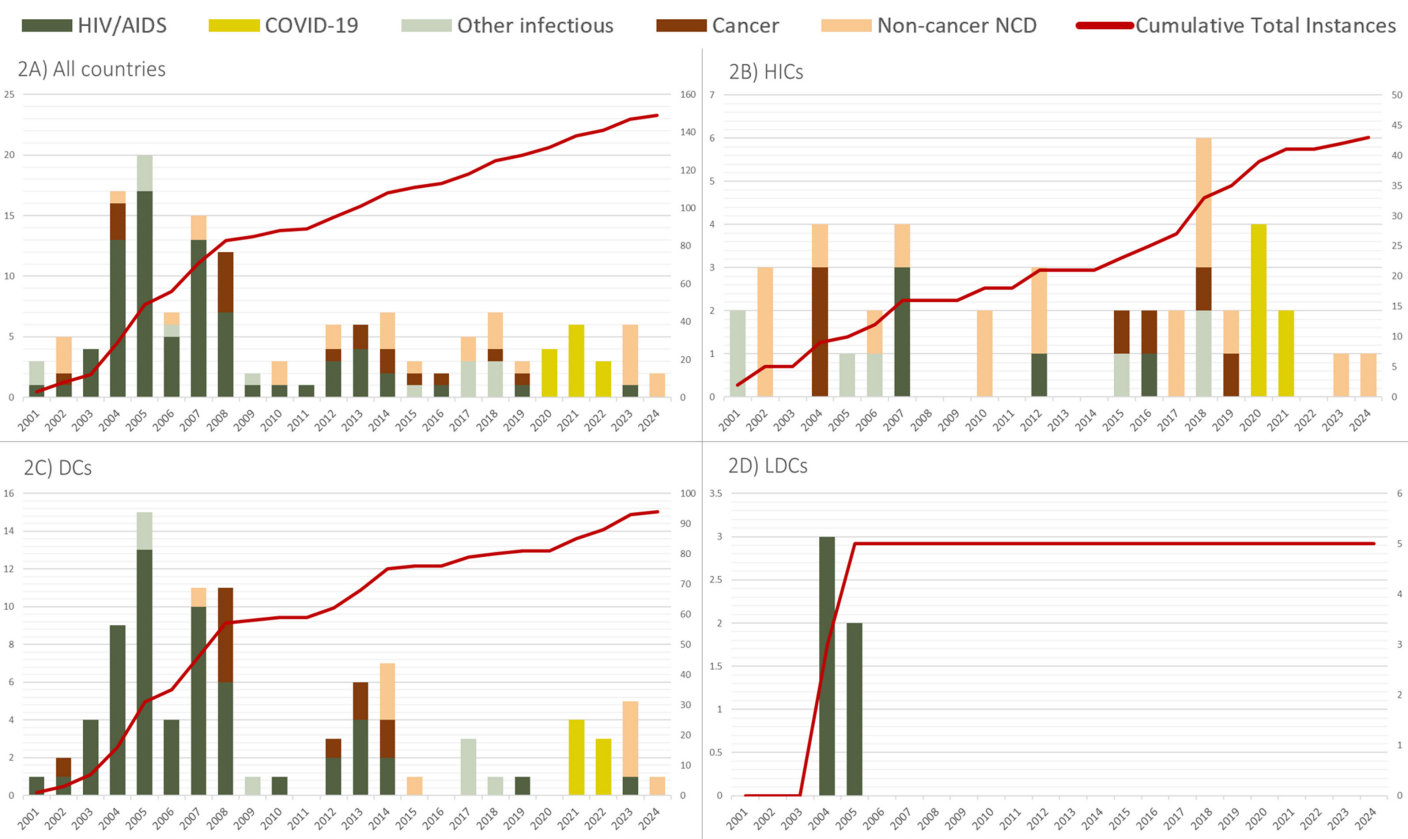
Instances of CLs by therapeutic indication and WTO classification: (A) All countries, (**B**) HICs, (**C**) DCs, (**D**) LDCs, 2001–2024. Note that y-axis scales differ for each WTO Country classification. CL, compulsory licensing; DCs, developing countries; HICs, high-income countries; LDCs, least-developed countries.

Figure 2A–D shows instances of CLs from 2001 to 2024, by WTO country classification and categorised by therapeutic indication.

#### A shift towards compulsory licences for NCDs

Before 2016, most CL instances were for HIV/AIDS treatments in DCs which do not benefit from the aforementioned LDC transition measure. However, since 2016, very few CLs have been issued worldwide for this disease (see figure 2A). Most CL instances globally are for NCDs such as cancer, cystic fibrosis and spinal muscular atrophy (SMA), with an important focus on COVID-19-related products between 2020 and2023.

Since 2010, the rate of CL instances for medicines to treat rare diseases has increased. For example, CF Transmembrane Conductance Regulator products for cystic fibrosis were the subject of six CL instances between 2019 and 2024 (see box 3). SMA is another example of a disease that affects few, the treatments for which are often prohibitively expensive (see the ‘Discussion’ section).

Box 3 **Cystic fibrosis (CF) and CFTR modulator therapies**

CF is a progressive genetic condition with a worldwide prevalence of over 150 000 people, making it one of the most common fatal genetic disorders.^59^ It is caused by a faulty protein that affects the lungs, digestive tract and other organs.^60^ There is significant variation in the clinical phenotype of CF patients, but as a population, the life expectancy for CF patients has historically been poor in HICs as well as DCs and LDCs.^61^

In 2012, a new class of medicines was approved for sale in the US market: CFTR modulator therapies would go on to drastically improve the mortality and morbidity of patients living with CF who were medically eligible for (and could access) them.^62^ The life expectancy of the median CF patient in the US was expected to live for an additional 23 years in 2021 vs 1996.^63^

Vertex Pharmaceuticals is a corporation with a portfolio of (CF) products ^64^ that holds the number 12 spot in terms of market value of all pharmaceutical companies.^65^ It is the sole IP holder of the CFTR medicines. As such, it has been able to charge hundreds of thousands of dollars per patient per year ^66^ in many HICs and reap profits in the tens of billions.^67^ Estimates of production costs with robust generic competition are 90% less than the current list price.^68^ The IP for these medications holds significant value for Vertex and it has refused to grant any VLs for supply to DCs or LDCs. The worldwide coverage of patients with triple therapy, the current gold standard of treatment, is estimated to be as low as 12%.^59^

The ML&P database holds six CL instances relating to CFTR modulators from the last 5 years, five of which were instigated by civil society campaigns for improved access. Two of these campaigns resulted in price discounts. The other instances remain pending.

CFTR, CF Transmembrane Conductance Regulator; DCs, developing countries; IP, intellectual property; LDCs, least-developed countries; VLs, voluntary licensing.

#### Continued use of CLs for infectious diseases

Although the vast majority of HIV CL instances are in DCs and LDCs, three CLs for these medicines were requested or granted in HICs. Two of these were in the USA (2007, 2012) and one in Germany (2016). The US instances, although not implemented, were a direct response to anti-competitive practices or price gouging. The German instance (raltegravir) was sought and executed in response to a patent infringement suit that threatened the supply of the product to the German public. The final instance is an Article 31bis case for export (see online supplemental annex S4).

Of the 13 CL instances for COVID-19 therapeutics, six were in HICs (one of which was a vaccine for export under TRIPS Article 31bis). Moreover, CL instances to address infectious diseases have been reported in HICs and DCs for avian influenza (four instances for oseltamivir) and hepatitis C (six instances for sofosbuvir).

### Section 3: outcomes of recorded instances of TRIPS flexibilities

**Figure 3:**
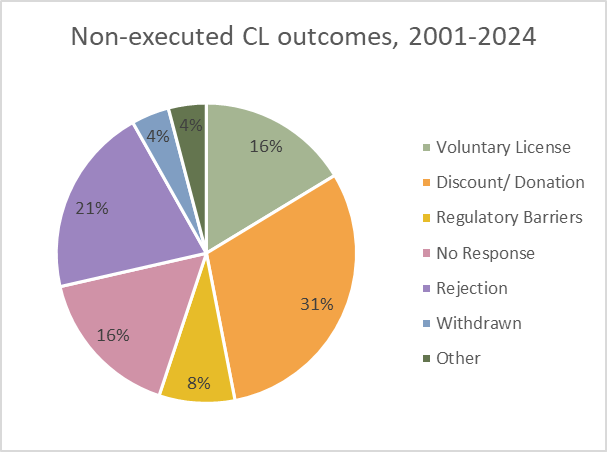
Non-executed CLs for all countries by outcome, 2021–2024. CLs, compulsory licensing.

Instances do not necessarily mean executed cases. Figure 3 represents the outcomes of non-executed CL instances, being: price discount/donation, VL, regulatory barriers and no response. See the Glossary (online supplemental file 2) for definitions of these outcomes.

Of the total 149 CL instances, 49 (32.9%) were not executed (see table 2 for breakdown by country classification). Of these, 23 (46.9%) were not executed due to an offer made by the patent holder that would increase access, such as a VL (16.3%), a price discount (28.6%) or a donation (2.0%).

**Table 2:** Outcomes of CLs by WTO Country Classification, 2001–2024

CL outcomes: 2001–2024 Summary				
WTO country classification	Executed *n (%)*	Non-executed *n (%)*	Pending (<5 years) *n (%)*	Total *n (%)*
Total	91 (61.1)	49 (32.9)	9 (6.0)	149 (100)
HIC	18 (41.9)	23 (53.5)	2 (4.7)	43 (28.9)
DC	61 (64.9)	26 (27.7)	7 (7.4)	94 (63.1)
LDC	5 (100)	0	0	5 (3.4)
Observer	7 (100)	0	0	7 (4.7)

DC, developing country; HIC, high-income country; LDC, least-developed country.

CL instances were more commonly executed in DCs (64.9%) than in HICs (41.9%). Of non-executed instances, VLs were more frequently the reason in DCs (23.1%) than in HICs (8.7%). Regulatory barriers and no response were more frequently the reasons for not executing a CL in HICs (13.0%, 21.7%) than in DCs (3.8%, 11.5%).

LDCs that have made use of the LDC pharmaceutical transition measure do not need to issue CLs for local supply of pharmaceutical products. Of the five instances of CLs in LDCs, one occurred without evidence of invoking the LDC transition measure and four were executed before the invocation of the LDC transition measure.

#### Applicants for CLs

Box 4 explains what is meant by ‘Government use’ licensing and highlights where more information can be found about its use by the USA.

Box 4 **Government use licences**

A ‘government use licence’ is a compulsory licence a government issues for its own use (‘Crown use’ in Commonwealth countries). The authorisation to use the patented invention can be used directly by the government, be given to a state agency or department, or to a third party including a private entity. In the Trade-Related Aspects of Intellectual Property Rights (TRIPS) Agreement, government use is referred to as ‘public non-commercial use’^69^ and can be issued without efforts to obtain a voluntary licensing and without needing to identify all applicable patents or patent holders. Government use can be particularly useful in the procurement of medicines by public institutions because it allows governments to run public tenders and purchase from the best-priced provider.

The USA has made extensive use of a particular form of government use licence, for pharmaceuticals and non-pharmaceuticals, written into its national law: Statute 28 US Code § 1498, which allows the US government to use or authorise third parties to use any US-granted patent, without permission or even notice to patent holders, so long as the use is ‘by or for’ the government. In many cases, the implementation of 28 US Code § 1498 is facilitated through the US Government’s Federal Acquisitions Regulations (FAR), specifically, the clause FAR 52.227–1, titled ‘authorization and consent’, which grants authorisation and consent to use any inventions covered by a US patent.^70^ This language falls most clearly under the provisions of TRIPS article 44.2, which is not one of the provisions examined in this study. Uses of this power are well documented in an online database hosted by Knowledge Ecology International.^71^

There were 32 recorded CL instances resulting from civil society campaigning (including by patient organisations) (see the Glossary (online supplemental file 2) for applicant definitions and online supplemental annex S4 for the data as a table). Slightly more CL instances resulting from a civil society campaign occurred in a DC than an HIC (43.8%, 56.3%). Of all 32 instances resulting from a civil society campaign, three were executed and seven (n=4 COVID-19 products; n=3 cystic fibrosis products) are still pending (<5 years old with no response). No instances led to a VL, and none were withdrawn. Of the non-executed CLs, eight (25%) led to a discount or donation of the product.

## Discussion

### Summary

This study provides the most comprehensive account to date of the use of compulsory licences and the LDC transition measure since the adoption of the Doha Declaration on TRIPS and Public Health in 2001. TRIPS flexibilities continue to be routinely used by WTO Members to facilitate access to medicines, with 199 reported instances between 2001 and 2024. Of these, most were instances of compulsory licensing (including public non-commercial use) (n=149) and LDC pharmaceutical transition measure (n=46), with some instances of use of the research exception (n=3) and parallel import (n=1). Five of the 149 CLs were Article 31bis instances. Of the CL instances, most were in DCs (n=94), but increasingly they have occurred in HICs (n=43). 61% (n=91) of CL instances were executed, with a higher proportion being executed in DCs than HICs. Since 2010, CLs have shifted to cover NCDs and increasingly, rare diseases (except from 2020 to 2023 when CLs addressed COVID-19 products). CL applicants were mostly governments, most frequently the US and Thailand, and some applications were the result of civil society campaigns (n=32, 21.4% of CLs).

Of the 32.9% of CL instances that were not executed, 30.6% (n=15) were abandoned because of a price discount offer, including one managed entry agreement, or a donation by the originator company. Such pricing and access offers are evidence of the indirect strong incentive effect of CLs in reducing price and improving access, driven by the wish of the rights holder to avoid the issuance of a CL.^39^

### Evolution and trends in TRIPS flexibility use

A previous study identified 144 instances of patent-related TRIPS flexibility use.^10^ Comparing the present findings to what was known in 2016 reveals seven important trends in national management of IP for access to medicines.

First, since 2016, there have been 36 new instances of CL (2001–2016: 113 instances; 2017–2024: 36 instances). Most of the instances since 2016 have not been executed (2001–2016: 69.9% executed; 2017–2024: 33.3% executed). 25% of cases since 2017 are categorised as pending. Instances have occurred at the approximate same frequency since 2010, but fewer are being executed.

Second, this study confirms that since 2009, no countries reported using the LDC pharmaceutical transition measure. This finding points to the fact that there are no formal notification requirements (and so instances here may represent the first of multiple uses of this right); however, publicly invoking the right to use the LDC transition measure helps assure suppliers that they do not risk patent infringement. Additionally, the need for the LDC transition measure in procurement has likely been reduced by the execution of VLs. In particular, execution of VLs for HIV products facilitated by the Medicines Patent Pool (established in 2010), whose licences always cover all LDCs. It is worth noting that the presence of a VL does not force a country to enforce a patent (eg, as allowed by the LDC transition measure), or preclude from using a CL should this offer better conditions.

Third, the use of Article 31bis, the special CL for export, remains sporadic with five instances (and only one executed) since 2001. This is the first global, longitudinal overview of the use of Article 31bis, not previously reported elsewhere. Its sporadic use can be attributed to easier alternative pathways (eg, regular CL to import generic medicines from countries where they are not patented) and the legal and procedural complexity of the Article 31bis mechanism at the national level. For example, in 2021 Canada received a request to allow the production of a COVID-19 vaccine for export but it could not be granted because the product was not listed in the Canadian Patent Act schedule that would allow Health Canada (national regulator) to evaluate the product. Such evaluation is a condition in Canadian law (not in the TRIPS Agreement) for granting a compulsory licence for export. Requests for CL to produce oseltamivir, imatinib and COVID-19 vaccines in Canada for export also never cleared this hurdle. The single executed instance of an Article 31bis measure to export generic ARVs produced in Canada to Rwanda took nearly 4 years.^40^

Fourth, this study shows an increase in the proportion of compulsory licensing instances in HICs, from 15% of all instances between 2005 and 2014 to 54% of all instances between 2015 and 2024. This is partly because of demands by patient interest groups made to their governments to take action to provide access to essential treatments for life-threatening diseases, such as cancer treatments and treatments for rare diseases. Elected officials, medical professional associations and other civil society organisations often support these requests. Further, the COVID-19 pandemic has spurred a renewed interest in the use of compulsory licensing in HICs.

Fifth, compulsory licensing for NCDs has increased, likely due to the shifting disease burden and rising treatment costs.^11 12^

Sixth, although total CL use for HIV/AIDs medicines has diminished, CLs are still a priority measure for access to these medicines in some countries (eg, dolutegravir CL, Colombia 2023). This is likely because upper-middle-income countries are often excluded from the sales territory of VLs.^41^ However, some VLs^42^ and all Medicines Patent Pool licences allow the licensees to supply to excluded territories (despite the existence of a patent in the manufacturing country) if such supply does not infringe on patent rights in the importing country. This is, for example, the case when no valid patents are granted or when a compulsory licence is issued.^41^ This can improve availability/affordability, particularly in places without sufficient national manufacturing capabilities/capacity.

Seventh, this study demonstrates that the use of TRIPS flexibilities to facilitate access to treatments for rare diseases has risen. For example, SMA affects worldwide 1/10 000 live births,^43^ yet two medicines to treat it have been the subject of two CL instances (2018, 2025). The latter is not included in the results as it falls outside the period of 2001–2024. The 2025 instance was executed in India to allow a generic producer offering the SMA treatment risdiplam to continue to do so for the duration of a patent infringement suit.^44^

### The future of TRIPS flexibilities

The increased focus on access to treatment for NCDs, coupled with the often high pricing of such treatments, will likely continue to spur interest in the use of TRIPS flexibilities. Since 2005, the TRIPS Agreement has been fully implemented at the national level by WTO Members,^45^ including countries that were the traditional manufacturing base for generic medicines prior to the TRIPS Agreement (eg, India). Therefore, sources of generic production of new essential medicines risk drying up except for products for which patent licences are available or TRIPS flexibilities are used.^46^ Those can be made available on a voluntary basis, for example, through the Medicines Patent Pool or directly by companies in bilateral agreements with generic producers, or on a non-voluntary basis by governments using TRIPS flexibilities. Companies show a reluctance to offer patent licences for NCD treatments. This, coupled with the crisis in global health funding and foreign aid cuts, may encourage governments to seek non-voluntary measures to ensure access to new essential medicines for their populations. Using non-voluntary measures will require clearing up current barriers to the effective use of TRIPS flexibilities.^47^

Barriers to the effective use of TRIPS flexibilities can be of a legal, political or practical nature. Several of the documented instances have been extremely politically fraught domestically and/or internationally. For example, in 2016, Colombia faced significant political pressure from the US and Switzerland over its move to issue a CL for imatinib,^48^ up to and including the US threat to withdraw from Colombia’s peace process.^49^ If national legislation is needlessly complex or demands decision-making at the highest level, the decision can be at higher risk of politicisation. Some HICs have opted out of using TRIPS Article 31bis for importation, which means they are not eligible to import medicines manufactured under a CL in another country, even in an emergency.^50^

The absence of generic production capacity and/or market prospects for the product in question can form practical barriers to the use of CL. This can have significant consequences in LDCs that depend on such production for imports.

### Strengths and limitations

This study presents the most comprehensive review of the uses of CLs and the LDC transition measure between 2001 and 2024. A key strength of this paper is its identification of previously unreported cases, which demonstrates the utility of our approach. To address gaps in the legal and empirical literature, this paper draws on the ML&P Database (which also includes information from governmental archives) to maximise the instances of CLs and LDC transition measures included. In the present study, only 16 new instances of TRIPS flexibilities were identified and added to the ML&P Database, which is the global reference repository for instances of TRIPS flexibilities.^51^,^53^ This suggests the Database is largely complete with all known reported instances of traceable TRIPS flexibility instances in the last 25 years.

However, despite the extensive scope of this review, it is possible that additional CL instances remain unidentified, particularly those not documented in public sources or those reported in a non-English language. We minimised this risk by translating all non-English documents retrieved through the search and screening them in consultation with experts fluent in the relevant languages (eg, Spanish, Ukrainian). Moreover, we also verified each reported instance against other sources (eg, allowing us to identify and exclude cases mistakenly reported by others as a TRIPS Flexibility). As there are no notification requirements for the use of the LDC transition measure, instances here track the known (first) invocations of the right to use this measure, meaning repeated uses are not captured. Unverified instances (n=23) were excluded from the results, which enhances the validity of our data, and that of the ML&P Database. Finally, we categorised each instance using clearly defined variables to promote the objectivity and reproducibility of the analysis.

### Implications for law and policy

WTO rules and in particular the TRIPS Agreement offer legal opportunities to use flexibilities in patent law to facilitate access to health products. However, these flexibilities must be implemented at the national level and included in national law in a manner that makes them easy to use. CL provisions need to include measures to waive exclusive rights in undisclosed information if such information is necessary to give effect to the CL. Data and market exclusivity, when provided for in medicines legislation, need to include a waiver in cases of CL.^54^ In the future, the special provision for CL for export (Article 31bis) may become more relevant; however, this will only be effective if the rules are implemented in a simplified manner and the exporting country responds swiftly to a request from another nation with insufficient production capacity. To have effective national implementation of TRIPS flexibilities, it is essential that HICs end their pursuit of TRIPS-plus provisions in trade agreements and desist from exerting pressure on other countries to refrain from adopting or utilising TRIPS flexibilities.

Private entities should give preference to licensing through the MPP and refrain from entering into VL agreements that curtail the use of TRIPS flexibilities by not allowing licensees to supply in countries where there are no or no longer patent barriers to such supply.

Considering the increase of CL instances in HICs, it may be advisable for HICs to opt back into the TRIPS Article 31bis mechanism as importers, which will ensure a broader range of suppliers in case of need, for example, during a pandemic.

The data from this study should inform a 30-year review of the TRIPS Agreement, which has been proposed at the WTO by Colombia and is under consideration.^55^ These data and findings can serve as a basis to assess national implementation and proposals for strengthening the public interest objectives of the TRIPS Agreement.^56^

### Implications for research

These findings, alongside the ML&P Database, offer valuable insights for future research on domestic patent law and access to medicines. First, although most countries have national law permitting the use of TRIPS Flexibilities, future research could examine what political, institutional or economic factors determine whether a country uses such flexibilities, and in which situations. Second, building on the work done by WIPO,^47^ future research could clarify the (legal, political and other) barriers to the domestic implementation of TRIPS Flexibilities. Third, greater attention should be given to exploring the possible indirect effects of CLs on medicines availability and affordability, the domestic health system and population health. Fourth, as only three of the 32 CL requests made by civil society organisations have led to a granted CL, future research should investigate the determinants of the success of civil society campaigns in securing the execution of a TRIPS Flexibility. Fifth, evaluative research should continue to build the body of knowledge about the direct impact of TRIPS flexibilities, and specifically CLs, on medicine availability, affordability and health outcomes in specific countries.

## Conclusions

The use of TRIPS flexibilities to facilitate access to medicines (including vaccines) has been truly global over the quarter-century since the Doha Declaration on TRIPS and Public Health. Compulsory licensing instances occur frequently in countries of a range of economic positions and are executed in most cases. Importantly, in those cases where CLs are not executed, many of them are successful in driving alternative access measures such as VLs, price reductions and donations.

As new medicines continue to be marketed at ever-increasing prices, the global burden of disease shifts and new public health crises threaten the supply of medicines and vaccines in HICs as well as DCs and LDCs, TRIPS flexibilities continue to play a vital role in ensuring that new essential medicines are available and affordable. They are likely to continue to do so in a future where geopolitical forces have drastically altered the financing structures of medicines provision in DCs and LDCs and medicines prices become unaffordable even in the richest HICs.

## Supplementary material

10.1136/bmjgh-2025-021481 online supplemental file 1

10.1136/bmjgh-2025-021481 online supplemental file 2

## Data Availability

Data are available in a public, open access repository.
