# Supplementary material for: TRIPS flexibilities help change policy and practice to increase access to medicines: evidence from 2001 to 2024
Source: BMJ Glob Health. 2026 Jan 28;11(1):e021481. doi: 10.1136/bmjgh-2025-021481 (PMC12853504; doi:10.1136/bmjgh-2025-021481)
Supplement: online supplemental file 1 [file bmjgh-11-1-s001.docx]

# TRIPS Flexibilities help change policy and practice to increase access to medicines: Evidence from 2001-2024

## Supplementary Annex

### Annex S1: Search Strings

EMBASE VIA OVID 08/07/2024

1. (pharmaceutical* or drug* or medication* or "medical product*" or "medicinal product*").af.

2. ("compulsory licen*" or "public noncommercial" or "public non-commercial" or "government* use").af.

3. ("least-developed countries pharmaceutical transition measure" or "LDC transition*" or "LDC pharma* transition*" or "LDC waiver").af.

4. "parallel import*".af.

5. ("research exception" or "patent exception").af.

6. 2 or 3 or 4 or 5

7. 1 and 6

8. ("art.31*" or "art. 31*" or "art 31*" or "article 31*").af.

9. ("para.7*" or "para. 7*" or "para 7*" or "paragraph 7*").af.

10. ("para.5*" or "para. 5*" or "para 5*" or "paragraph 5*").af.

11. ("art.30*" or "art. 30*" or "art 30*" or "article 30*").af.

12. ("TRIPS" or "trade related aspects of intellectual property rights" or "trade-related aspects of intellectual property rights" or "Doha").af.

13. 8 or 9 or 10 or 11

14. 13 adj10 12

15. 1 and 14

16. 7 or 15

391 RESULTS 2001-onwards

MEDLINE VIA OVID 08/07/2024

1. (pharmaceutical* or drug* or medication* or "medical product*" or "medicinal product*").af.

2. ("compulsory licen*" or "public noncommercial" or "public non-commercial" or "government* use").af.

3. ("least-developed countries pharmaceutical transition measure" or "LDC transition*" or "LDC pharma* transition*" or "LDC waiver").af.

4. "parallel import*".af.

5. ("research exception" or "patent exception").af.

6. 2 or 3 or 4 or 5

7. 1 and 6

8. ("art.31*" or "art. 31*" or "art 31*" or "article 31*").af.

9. ("para.7*" or "para. 7*" or "para 7*" or "paragraph 7*").af.

10. ("para.5*" or "para. 5*" or "para 5*" or "paragraph 5*").af.

11. ("art.30*" or "art. 30*" or "art 30*" or "article 30*").af.

12. ("TRIPS" or "trade related aspects of intellectual property rights" or "trade-related aspects of intellectual property rights" or "Doha").af.

13. 8 or 9 or 10 or 11

14. 13 adj10 12

15. 1 and 14

16. 7 or 15

205 RESULTS 2001-onwards

LEXISNEXIS CASES 1

(pharmaceutical* or drug* or medication* or "medical product*" or "medicinal product*") and ("compulsory licen*" or "public noncommercial" or "public non-commercial" or "government* use" or "least-developed countries pharmaceutical transition measure" or "LDC transition*" or "LDC pharma* transition*" or "LDC waiver" or "parallel import*" or "research exception" or "patent exception")

Filters:

- Cases
- IP tag
- 01/01/2001 – 31/12/2024

328 RESULTS

LEXISNEXIS CASES 2

(pharmaceutical* or drug* or medication* or "medical product*" or "medicinal product*") and (("art.31*" or "art. 31*" or "art 31*" or "article 31*" or "para.7*" or "para. 7*" or "para 7*" or "paragraph 7*" or "para.5*" or "para. 5*" or "para 5*" or "paragraph 5*" or "art.30*" or "art. 30*" or "art 30*" or "article 30*") w/10 ("TRIPS" or "trade related aspects of intellectual property rights" or "trade-related aspects of intellectual property rights" or "Doha"))

Filters:

- Cases
- IP tag
- 01/01/2001 – 31/12/2024

48 RESULTS

### Annex S2: PRISMA Flowchart


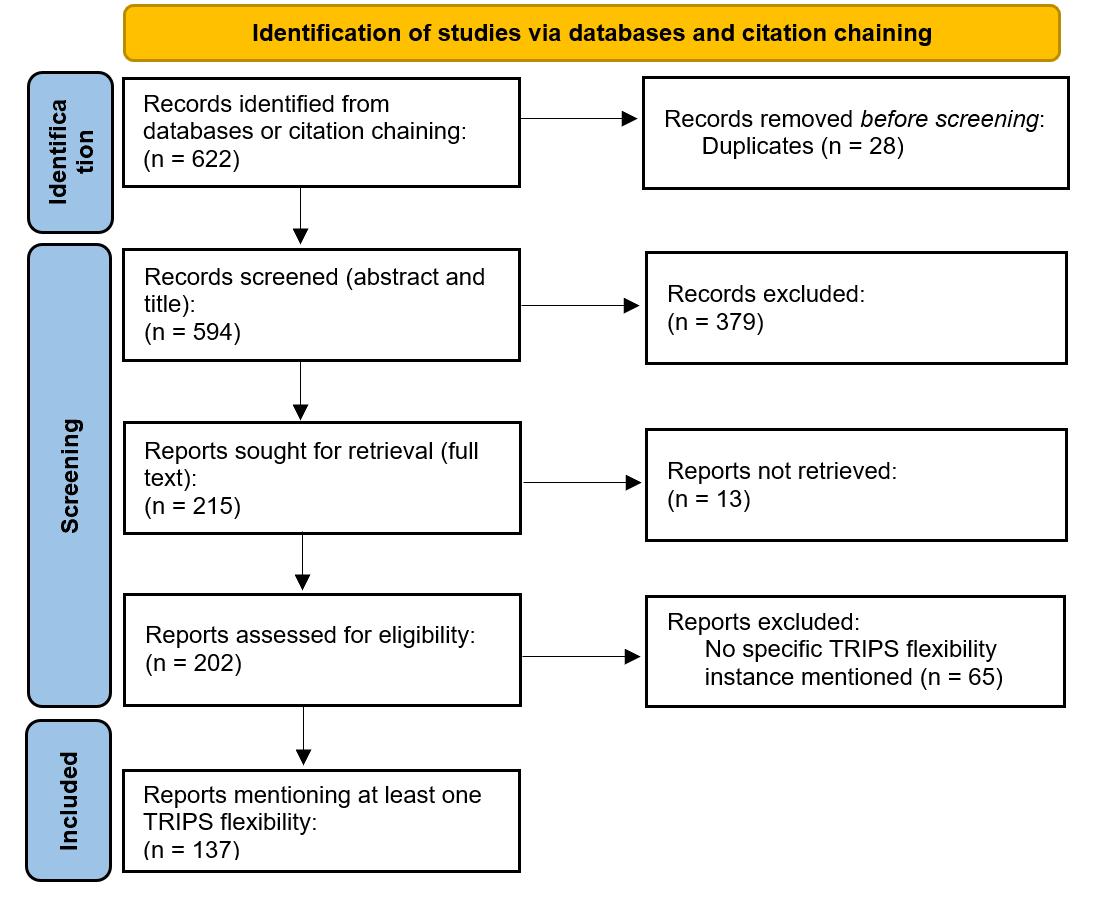


### Annex S3: Included papers

| No. | Title | Year | Authors |
| --- | --- | --- | --- |
| 1 | Joined opinion of Mr Advocate General Gulmann delivered on 12 July 2001. Merck, Sharp & Dohme GmbH v Paranova Pharmazeutika Handels GmbH. Reference for a preliminary ruling: Oberlandesgericht Wien - Austria. Case C-443/99. Boehringer Ingelheim KG, Boehringer Ingelheim Pharma KG, Glaxo Group Ltd, The Wellcome Foundation Ltd, SmithKline Beecham plc, Beecham Group plc, SmithKline & French Laboratories Ltd and Eli Lilly and Co. v Swingward Ltd and Dowelhurst Ltd. Reference for a preliminary ruling: High Court of Justice (England & Wales), Chancery Division - United Kingdom. Case C-143/00. Trade marks - Directive 89/104/EEC - Article 7(2) - Exhaustion of the rights conferred by the trade mark - Pharmaceutical products - Parallel importation - Repackaging of the trade-marked product. ECLI:ECLI:EU:C:2001:412 EU Cases | 2001 |  |
| 2 | BOEHRINGER INGELHEIM PHARMA GmbH & CO KG Pursuers against MUNRO WHOLESALE MEDICAL SUPPLIES LTD Defenders 2003 Scot (D) 1/9 Scottish Court Opinions | 2003 |  |
| 3 | Opinion of Mr Advocate General Jacobs delivered on 28 October 2004. Synetairismos Farmakopoion Aitolias & Akarnanias (Syfait) and Others v GlaxoSmithKline plc and GlaxoSmithKline AEVE. Reference for a preliminary ruling: Epitropi Antagonismou - Greece. Admissibility - Meaning of court or tribunal of a Member State - Abuse of a dominant position - Refusal to supply pharmaceutical products to wholesalers - Parallel trade. Case C-53/03. ECLI:ECLI:EU:C:2004:673 EU Cases | 2004 |  |
| 4 | Opinion of Advocate General Sharpston delivered on 9 October 2008. The Wellcome Foundation Ltd v Paranova Pharmazeutika Handels GmbH. Reference for a preliminary ruling: Oberster Gerichtshof - Austria. Trade marks - Pharmaceutical products - Repackaging - Parallel imports - Substantial change in appearance of the packaging - Obligation of prior notice. Case C-276/05. ECLI:ECLI:EU:C:2008:547 EU Cases | 2008 |  |
| 5 | Opinion of Mr Advocate General Bot delivered on 12 May 2011. Orifarm A/S and Others (C-400/09) and Paranova Danmark A/S and Paranova Pack A/S (C-207/10) v Merck Sharp & Dohme Corp. and Merck Sharp & Dohme BV and Merck Sharp & Dohme. References for a preliminary ruling: HÃ¸jesteret - Denmark. Trade marks - Directive 89/104/EEC - Article 7(2) - Pharmaceutical products - Parallel imports - Repackaging of the product bearing the trade mark - New packaging indicating as the repackager the holder of the marketing authorisation on whose instructions the product was repackaged - Physical repackaging carried out by a separate undertaking. Joined cases C-400/09 and C-207/10. ECLI:ECLI:EU:C:2011:293 EU Cases | 2011 |  |
| 6 | Merck Sharp & Dohme Corp v Clonmel Healthcare Ltd [2018] IECA 177 Irish Judgments | 2018 |  |
| 7 | Bayer Intellectual Property GMBH v Kohlpharma GMBH Case Overview | 2022 |  |
| 8 | Developing nations and the compulsory license: maximizing access to essential medicines while minimizing investment side effects | 2009 | Bird, R. C. |
| 9 | Lost in translation: the Canadian access to medicines regime from transnational activism to domestic implementation | 2010 | Bubela, T. and Morin, J. F. |
| 10 | The Legislative Approach and System Improvement of China's Compulsory Licensing for Drug Patents | 2021 | Cao, Z. and Chen, Y. and Jiang, W. and Li, W. |
| 11 | Compulsory licensure: the case of Cipro and beyond | 2002 | Chakrabarty, A. M. |
| 12 | Trips Agreement Article 31(B): The Need for Revision | 2016 | Effingham, A. M. |
| 13 | The Brazilian experiment: HIV drugs for all | 2009 | Flaer, P. J. and Younis, M. Z. |
| 14 | Compulsory licensing to regulated licensing: effects on the conflict between innovation and access in the pharmaceutical industry | 2003 | Gillat, A. |
| 15 | An exploration of compulsory licensing as an effective policy tool for antiretroviral drugs in India | 2013 | Jain, D. and Darrow, J. J. |
| 16 | Realizing Public Rights Through Government Patent Use | 2021 | Kapczynski, A. |
| 17 | Drug Use Evaluation of Letrozole in Breast Cancer Patients at Regional Cancer Hospitals in Thailand | 2015 | Ketkaew, C. and Kiatying-Angsulee, N. |
| 18 | Access to medicines and domestic compulsory licensing: learning from Canada and Thailand | 2011 | Kuek, V. and Phillips, K. and Kohler, J. C. |
| 19 | Intellectual property rights and the Canadian pharmaceutical marketplace: where do we go from here? | 2005 | Lexchin, J. |
| 20 | Potential conflict between TRIPS and GATT concerning parallel importation of drugs and possible solution to prevent undesirable market segmentation | 2011 | Lo, C. F. |
| 21 | Compulsory licensing in Canada and Thailand: comparing regimes to ensure legitimate use of the WTO rules | 2009 | Lybecker, K. M. and Fowler, E. |
| 22 | Compulsory licenses: a tool to improve global access to the HPV vaccine? | 2009 | Maybarduk, P. and Rimmington, S. |
| 23 | Modeling HIV/AIDS drug price determinants in Brazil: is generic competition a myth? | 2011 | Meiners, C. and Sagaon-Teyssier, L. and Hasenclever, L. and Moatti, J. P. |
| 24 | Rational use of medicines - Indian perspective! | 2015 | Mohanta, G. P. and Manna, P. K. |
| 25 | Finding flaws: the limitations of compulsory licensing for improving access to medicines--an international comparison | 2008 | Ng, E. and Kohler, J. C. |
| 26 | Not taking, just borrowing: government use of patented drugs | 2002 | Ossorio, P. N. |
| 27 | Affordability versus innovation: Is compulsory licensing the solution? | 2019 | P and ey, E. and Paul, S. B. |
| 28 | Comment: compulsory licensing of patented pharmaceutical inventions: evaluating the options | 2009 | Reichman, J. H. |
| 29 | Response to commentaries : Resnik, D. B. and K. A. DeVille. 2002. "Bioterrorism and patient rights: 'compulsory licensure' and the case of Cipro." The American Journal of Bioethics 2(3): 29-39 | 2002 | Resnik, D. B. and DeVille, K. A. |
| 30 | Bioterrorism and patent rights: "compulsory licensure" and the case of Cipro | 2002 | Resnik, D. B. and DeVille, K. A. |
| 31 | Policies and programs to facilitate access to targeted cancer therapies in Thailand | 2015 | Sruamsiri, R. and Ross-Degnan, D. and Lu, C. Y. and Chaiyakunapruk, N. and Wagner, A. K. |
| 32 | Impact of the World Trade Organization TRIPS agreement on the pharmaceutical industry in Thailand | 2001 | Supakankunti, S. and Janjaroen, W. S. and Tangphao, O. and Ratanawijitrasin, S. and Kraipornsak, P. and Pradithavanij, P. |
| 33 | Data exclusivity exceptions and compulsory licensing to promote generic medicines in the European Union: A proposal for greater coherence in European pharmaceutical legislation | 2017 | t Hoen, E. F. M. and Boulet, P. and Baker, B. K. |
| 34 | Legal and policy foundations for global generic competition: Promoting affordable drug pricing in developing societies | 2015 | Zapatero Miguel, P. |
| 35 | How Much Time Before Attempting Compulsory Licensing of Pharmaceuticals? A Non-parametric Event History Model With P-splines | 2019 | Son, K. B. |
| 36 | Understanding of for whom, under what conditions and how the compulsory licensing of pharmaceuticals works in Brazil and Thailand: A realist synthesis | 2019 | Son, K. B. and Kim, C. Y. and Lee, T. J. |
| 37 | Compulsory Licensing of Pharmaceuticals in High-Income Countries: A Comparative Analysis | 2022 | Qunaj, L. and Kaltenboeck, A. and Bach, P. B. |
| 38 | Divergence and Convergence of Royalty Determinations between Compulsory Licensing under the TRIPS Agreement and Ongoing Royalties as an Equitable Remedy | 2020 | Shore, D. |
| 39 | The role of intellectual property rights on access to medicines in the WHO African region: 25 years after the TRIPS agreement | 2021 | Motari, M. and Nikiema, J. B. and Kasilo, O. M. J. and Kniazkov, S. and Loua, A. and Sougou, A. and Tumusiime, P. |
| 40 | Treatment advocate tactics to expand access to antiviral therapy for HIV and viral hepatitis C in low- to high-income settings: making sure no one is left behind | 2018 | Grillon, C. and Krishtel, P. R. and Mellouk, O. and Basenko, A. and Freeman, J. and Mendao, L. and Andrieux-Meyer, I. and Morin, S. |
| 41 | What is the impact of intellectual property rules on access to medicines? A systematic review | 2022 | Tenni, B. and Moir, H. V. J. and Townsend, B. and Kilic, B. and Farrell, A. M. and Keegel, T. and Gleeson, D. |
| 42 | Fighting Excessive Pharmaceutical Prices: Evaluating the Options | 2020 | den Exter, A. |
| 43 | Importance of the intellectual property system in attempting compulsory licensing of pharmaceuticals: a cross-sectional analysis | 2019 | Son, K. B. |
| 44 | A method for understanding generic procurement of HIV medicines by developing countries with patent protection | 2017 | Beall, R. F. and Attaran, A. |
| 45 | Pharmaceutical patents: reconciling the human right to health with the incentive to invent | 2020 | Khachigian, L. M. |
| 46 | A Critical Discourse Analysis of Intellectual Property Rights Within NAFTA 1.0: Implications for NAFTA 2.0 and for Democratic (Health) Governance in Canada | 2020 | Mohamed, F. A. and Chaufan, C. |
| 47 | Evaluating the Usefulness of Compulsory Licensing in Developing Countries: A Comparative Study of Thai and Brazilian Experiences Regarding Access to Aids Treatments | 2017 | Guennif, S. |
| 48 | Antiretroviral treatment, government policy and economy of HIV/AIDS in Brazil: is it time for HIV cure in the country? | 2019 | Benzaken, A. S. and Pereira, G. F. M. and Costa, L. and Tanuri, A. and Santos, A. F. and Soares, M. A. |
| 49 | Improving access to medicines: lessons from 10 years of drug reforms in China, 2009-2020 | 2022 | Mao, W. and Jiang, H. and Mossialos, E. and Chen, W. |
| 50 | Designing the global vaccine supply chain: balancing intellectual property rights with post COVID-19 vaccine equity | 2023 | Park, S. P. and Lee, H. J. and Yu, Y. and Lee, E. Y. J. and Park, Y. S. |
| 51 | Trade-Related Aspects of Intellectual Property Rights Flexibilities and Public Health: Implementation of Compulsory Licensing Provisions into National Patent Legislation | 2023 | Mc, Givern L. |
| 52 | The Bayh-Dole Act at 40: Accomplishments, Challenges, and Possible Reforms | 2022 | Sarpatwari, A. and Kesselheim, A. S. and Cook-Deegan, R. |
| 53 | AIDS and Africa. Introduction | 2002 | Kopelman, L. M. and van Niekerk, A. A. |
| 54 | Questions raised about whether compulsory licenses get best prices | 2015 | Owens, B. |
| 55 | Intellectual property rights, compulsory licensing and the TRIPS agreement: some ethical issues | 2003 | Schuklenk, U. |
| 56 | Affordable access to essential medication in developing countries: conflicts between ethical and economic imperatives | 2002 | Schuklenk, U. and Ashcroft, R. E. |
| 57 | The politics behind the implementation of the WTO Paragraph 6 Decision in Canada to increase global drug access | 2012 | Esmail, L. C. and Kohler, J. C. |
| 58 | The time for pharmaceutical compulsory licensing has expired | 2012 | Kuhn, R. and Beall, R. F. |
| 59 | Does reimportation reduce price differences for prescription drugs? Lessons from the European Union | 2008 | Kyle, M. K. and Allsbrook, J. S. and Schulman, K. A. |
| 60 | Drug prices and trends before and after requesting compulsory licenses: the Ecuadorian experience | 2019 | Ortiz-Prado, E. and Cevallos-Sierra, G. and Teran, E. and Vasconez, E. and Borrero-Maldonado, D. and Ponce Zea, J. and Simbana-Rivera, K. and Gomez-Barreno, L. |
| 61 | Asserting the primacy of health over patent rights: a comparative study of the processes that led to the use of compulsory licensing in Thailand and Brazil | 2014 | Rosenberg, S. T. |
| 62 | Compulsory licensing of generic drugs remains mired in quagmires | 2011 | Chami, G. and Wasswa-Kintu, S. |
| 63 | Government use licenses in Thailand: an assessment of the health and economic impacts | 2011 | Yamabhai, I. and Mohara, A. and Tantivess, S. and Chaisiri, K. and Teerawattananon, Y. |
| 64 | Panel: Canada's law on global access to affordable medicines | 2009 | Avafia, T. and Morrison, C. and Clark, B. and Kohler, J. C. |
| 65 | Steps forward, backward, and sideways: Canada's bill on exporting generic pharmaceuticals | 2004 | Elliott, R. |
| 66 | Will they deliver treatment access?: WTO rules and Canada's law on generic medicine exports | 2006 | Elliott, R. |
| 67 | First test of WTO mechanism for procuring generic medicines under compulsory licence, via Canada's Access to Medicines Regime | 2007 | Elliott, R. |
| 68 | [Compulsory licensing of efavirenz in Brazil in 2007: contextualization] | 2009 | Rodrigues, W. C. and Soler, O. |
| 69 | Civil society strategy for the compulsory licensing of lopinavir/ritonavir: the Brazilian case | 2008 | Vieira, M. F. and Reis, R. and Chaves, G. |
| 70 | Balancing public health, trade and intellectual monopoly privileges: recent Australian IP legislation and the TPPA | 2012 | Vines, T. and Crow, K. and Faunce, T. |
| 71 | Compulsory licenses and access to HIV/AIDS drugs | 2001 | Zuniga, J. M. and Wilder, R. |
| 72 | Initiatives to challenge patent barriers and their relationship with the price of medicines procured by the Brazilian Unified National Health System | 2016 | Scopel, C. T. and Chaves, G. C. |
| 73 | Canada's implementation of the Paragraph 6 Decision: is it sustainable public policy? | 2007 | Cohen-Kohler, J. C. and Esmail, L. C. and Cosio, A. P. |
| 74 | TRIPS to Where? A Narrative Review of the Empirical Literature on Intellectual Property Licensing Models to Promote Global Diffusion of Essential Medicines | 2021 | Mermelstein, S. and Stevens, H. |
| 75 | Canada's Access to Medicines Regime: Promise or Failure of Humanitarian Effort? | 2010 | Kohler, J. C. and Lexchin, J. and Kuek, V. and Orbinski, J. |
| 76 | Thailand and the compulsory licensing of efavirenz | 2007 | Steinbrook, R. |
| 77 | Battling chronic myeloid leukemia in a resource-constrained country: A case of public-private partnerships | 2023 | Tariq, R. and Fatima, I. and Shahid, M. H. and Tariq, S. and Niaz, F. and Hussain, S. M. |
| 78 | Trends in compulsory licensing of pharmaceuticals since the Doha Declaration: a database analysis | 2012 | Beall, R. and Kuhn, R. |
| 79 | Pandemics, antiviral stockpiles and biosecurity in Australia: what about the generic option? | 2006 | Lokuge, B. and Drahos, P. and Neville, W. |
| 80 | India to consider raft of new compulsory licences | 2014 | Bagcchi, S. |
| 81 | Improving access to medicines to reduce marketing and use of substandard and falsified medicines in Africa: Scoping review | 2024 | Sorato, M. M. and Davari, M. and Kebriaeezadeh, A. |
| 82 | [Constrained competition in parallel drug importation: the case of simvastatin in Germany, the Netherlands, and the United Kingdom] | 2007 | Costa-Font, J. and Kanavos, P. |
| 83 | Impact of the introduction of government use licenses on the drug expenditure on seven medicines in Thailand | 2012 | Mohara, A. and Yamabhai, I. and Chaisiri, K. and Tantivess, S. and Teerawattananon, Y. |
| 84 | Sustaining access to antiretroviral therapy in the less-developed world: lessons from Brazil and Thailand | 2007 | Ford, N. and Wilson, D. and Costa Chaves, G. and Lotrowska, M. and Kijtiwatchakul, K. |
| 85 | Brazilian policy of universal access to AIDS treatment: sustainability challenges and perspectives | 2007 | Greco, D. B. and Simao, M. |
| 86 | Solution to contentious issue of Article 31(f) of TRIPS agreement | 2008 | Janodia, M. D. and Sreedhar, D. and Ligade, V. S. and Udupa, N. |
| 87 | The economics of effective AIDS treatment in Thailand | 2007 | Over, M. and Revenga, A. and Masaki, E. and Peerapatanapokin, W. and Gold, J. and Tangcharoensathien, V. and Thanprasertsuk, S. |
| 88 | Patent litigation in India continues to throw up new challenges | 2016 | Reddy Thikkavarapu, P. |
| 89 | Trans-Pacific Partnership Agreement and Its Impact on Accessibility and Affordability of Medicines: A Meta-synthesis | 2017 | Yap, Y. Y. and Wong, C. P. and Lee, K. S. and Ming, L. C. and Khan, T. M. |
| 90 | Access to cancer medications in low- and middle-income countries | 2013 | Lopes Gde, L., Jr. and de Souza, J. A. and Barrios, C. |
| 91 | Access to critical medicines: When are compulsory licenses effective in price negotiations? | 2015 | Ramani, S. V. and Urias, E. |
| 92 | The case for compulsory licensing during COVID-19 | 2020 | Wong, H. |
| 93 | A one-time-only combination: Emergency medicine exports and the TRIPS agreement under Canada's access to medicines regime | 2010 | Weber, A. and Mills, L. |
| 94 | Kenya allows parallel importation of drugs | 2001 | Siringi, S. |
| 95 | Compulsory licensing often did not produce lower prices for antiretrovirals compared to international procurement | 2015 | Beall, R. F. and Kuhn, R. and Attaran, A. |
| 96 | Learning from practice: compulsory licensing cases and access to medicines | 2013 | Stirner, B. and Thangaraj, H. |
| 97 | Medicine procurement and the use of flexibilities in the Agreement on Trade-Related Aspects of Intellectual Property Rights, 2001-2016 | 2018 | t Hoen, E. F. and Veraldi, J. and Toebes, B. and Hogerzeil, H. V. |
| 98 | Indian health groups welcome country's first compulsory licence | 2012 | Mudur, G. |
| 99 | Access to hepatitis C virus treatment: Lessons from implementation of strategies for increasing access to antiretroviral treatment | 2018 | Assefa, Y. and Hill, P. S. and Williams, O. D. |
| 100 | Thailand: government issues compulsory licences for HIV/AIDS drugs | 2007 | Seim, L. |
| 101 | Research on the pharmaceutical intellectual property protection and supervision of pharmacy administration | 2017 | Xu, Z. and Chen, W. |
| 102 | Threat of compulsory licences could increase access to essential medicines | 2019 | Ooms, G. and Hanefeld, J. |
| 103 | Compulsory Licensing in Context of the Covid-19 Pandemic as a Tool for Ensuring the Balance between Rights-Holders' and Society's Interests | 2020 | Chepys, O. |
| 104 | Access to essential medicines for sexual and reproductive health care: the role of the pharmaceutical industry and international regulation | 2011 | Cottingham, J. and Berer, M. |
| 105 | The German Federal Supreme Court also granted a compulsory patent license for the first time in its history | 2017 | Komatani, T. S. |
| 106 | Compulsory licensing of patents | 2022 | Kumutha, N. and Amutha, N. and Venkatesh, G. S. |
| 107 | Parallel imports and innovation in an emerging economy: the case of Indian pharmaceuticals | 2012 | Mantovani, A. and Naghavi, A. |
| 108 | Health regulation and technological development: innovative strategies for accessing medicines in the SUS | 2021 | Rech, N. and Farias, M. R. |
| 109 | Compulsory licensing issues and trends in Asia | 2013 | Rungpry, S. K. |
| 110 | Learning from practice: compulsory licensing cases and access to medicines | 2012 | Stirner, B. |
| 111 | How can states provide affordable pharmaceuticals to the underserved? | 2006 | Zara, J. |
| 112 | Evolution of antiretroviral drug costs in Brazil in the context of free and universal access to AIDS treatment | 2007 | Nunn, A. S. and Fonseca, E. M. and Bastos, F. I. and Gruskin, S. and Salomon, J. A. |
| 113 | Expanding drug access in Brazil: lessons for Latin America and Canada | 2006 | Cohen, J. C. |
| 114 | Evidence-based decision-making in Asia-Pacific with rapidly changing health-care systems: Thailand, South Korea, and Taiwan | 2009 | Jirawattanapisal, T. and Kingkaew, P. and Lee, T. J. and Yang, M. C. |
| 115 | Compulsory Licenses for Cancer Drugs: Does Circumventing Patent Rights Improve Access to Oncology Medications? | 2016 | Bognar, Clfb and Bychkovsky, B. L. and Lopes, G. L., Jr. |
| 116 | Is Compulsory Licensing Bad for Public Health? Some Critical Comments on Drug Accessibility in Developing Countries | 2017 | Guennif, S. |
| 117 | Compulsory licensing of pharmaceuticals reconsidered: Current situation and implications for access to medicines | 2018 | Son, K. B. and Lee, T. J. |
| 118 | The link between publicly funded health care and compulsory licensing | 2002 | Hollis, A. |
| 119 | Compulsory licences for direct acting antiviral drugs for hepatitis C | 2016 | Cattaneo, A. and Maciocco, G. |
| 120 | Compulsory licensing of patents in India | 2016 | Chaudhry, R. |
| 121 | The welfare impact of parallel imports: a structural approach applied to the German market for oral anti-diabetics | 2014 | Duso, T. and Herr, A. and Suppliet, M. |
| 122 | A survey of the syntheses of active pharmaceutical ingredients for antiretroviral drug combinations critical to access in emerging nations | 2008 | Pinheiro Edos, S. and Antunes, O. A. and Fortunak, J. M. |
| 123 | Drug policy and administration affecting quality of life of the poor in Thailand | 2011 | Prutipinyo, C. and Sirichotiratana, N. |
| 124 | Parallel imports in the enlarged EU: the specific mechanism | 2013 | Stothers, C. |
| 125 | Canada and access to medicines in developing countries: intellectual property rights first | 2013 | Lexchin, J. |
| 126 | Bayer challenges India's first compulsory licence for generic version of cancer drug | 2012 | Arie, S. |
| 127 | World Trade Organisation reaches agreement on generic medicines. New deal will make it easier for poorer countries to import cut-price generic drugs made under compulsory licensing | 2003 | Kapp, C. |
| 128 | 5-Year Evaluation of drug utilization, costs and outcomes of chronic hepatitis c treatment in malaysia: Picture of precompulsory licensing era | 2019 | Rahman, M. F. A. B. D. and Hassan, M. R. A. and Keat, C. H. and Omar, H. and Md Said, R. and Yue, C. L. and Ibrahim, N. R. W. and Mutalib, N. A. |
| 129 | Access of developing countries to essential drugs: a process of equity. [French] | 2001 | Campion, M. D. |
| 130 | Achieving equitable access to CFTR modulators: every patient in every country | 2023 | Guo, J. and King, I. and Hill, A. |
| 131 | Analysis of India compulsory license system of drug patent and the enlightenment to China. [Chinese] | 2016 | Yu, C. H. and Tian, K. and Yu, X. Y. |
| 132 | Antiretroviral drugs in developing countries: Myth or reality?. [French] | 2003 | Calmy, A. and Hirschel, B. |
| 133 | Antiretroviral drugs: Analysis of Ukrainian pharmaceutical market and intellectual property | 2017 | Litvinova, E. V. and Posilkina, O. V. |
| 134 | Application of Compulsory Licensing in the Context of the Covid-19 Coronavirus Pandemic | 2021 | Serohina, N. and Pikhurets, O. and Iasechko, S. and Yevlakhova, E. and Lytvyn, S. and Miroshnykov, I. |
| 135 | Brazil's AIDS controversy: Antiretroviral drugs, breaking patents, and compulsory licensing | 2005 | Quintanilha Marques, U. R. and Santos Guimaraes, V. and Sternberg, C. |
| 136 | Brinkmanship and compulsory licensing policy lessons from Brazil | 2007 | Wasunna, A. A. |
| 137 | Competing Responses to Global Inequalities in Access to COVID Vaccines: Vaccine Diplomacy and Vaccine Charity Versus Vaccine Liberty | 2022 | Sparke, M. and Levy, O. |
| 138 | Compulsory licence and access to medicines: Economic savings of efavirenz in Brazil | 2012 | Viegas Neves Da Silva, F. and Hallal, R. and Guimaraes, A. |
| 139 | Compulsory licences on pharmaceutical patents in India: A short article | 2017 | Rathod, S. K. |
| 140 | Compulsory licenses for medicines | 2015 | Hoen, E. and Bermudez, J. |
| 141 | Compulsory licenses for non-communicable diseases: Implications for pharmaceutical pricing in low-income countries | 2012 | Wasserman, M. and Priest, V. L. |
| 142 | Compulsory licenses: The authors reply | 2015 | Beall, R. F. and Attaran, A. and Kuhn, R. |
| 143 | Compulsory licensing in pharmaceutical industry: Current state of affairs and prospects | 2021 | Gaydin, T. Y. and Rozhnova, S. A. |
| 144 | Compulsory licensing of chronic disease pharmaceuticals in Thailand | 2013 | Thanitcul, S. and Braslow, M. L. |
| 145 | Compulsory licensing of drug products in developing countries | 2015 | Saroha, S. and Kaushik, D. and N and a, A. |
| 146 | Compulsory licensing of pharmaceutical products for export to countries with public health problems: Legal myth or legal reality?. [French] | 2011 | Mascret, C. |
| 147 | Compulsory licensing: Procedural requirements under the TRIPS agreement | 2016 | Desai, M. A. |
| 148 | Compulsory pharmaceutical patent licensing in Brazil: The controversy of public interest | 2013 | Rodrigues Da Silva, C. and Galvao De Botton, L. |
| 149 | Costs, Challenges and Outcomes of Chronic Hepatitis C Treatment with Direct-Antiviral Agents from Provider-Based Perspective in Malaysia: Before the Era of Compulsory Licensing | 2018 | Rahman, M. F. A. and Said, R. M. and Omar, H. and Mutalib, N. and Chan, H. K. and Wan Ibrahim, N. R. and Chan, L. Y. and Hassan, M. R. A. |
| 150 | COVID-19, IP and access: Will the current system of medical innovation and access to medicines meet global expectations? | 2021 | Gurgula, O. and Lee, W. H. |
| 151 | Developments in parallel import, current decisions - Part I. [German] | 2001 | Kleist, H. |
| 152 | Developments in parallel import. Current decisions: Part II. [German] | 2001 | Kleist, H. |
| 153 | Does the legal regulation of promoting pharmaceutical parallel imports helps to create savings for the statutory health insurance (GKV) in Germany? | 2015 | Leisten, M. and Brakmann, D. and Munchberg, F. and Wiberny, S. |
| 154 | Economical issue of new drug use for patients | 2012 | Lusaya, D. G. |
| 155 | EPS10.10 Elexacaftor/tezacaftor/ivacaftor remains inaccessible for people with cystic fibrosis in low- and middle-income countries: how can this be solved? | 2024 | Guo, J. and King, I. and Hill, A. |
| 156 | Erratum: Learning from practice: Compulsory licensing cases and access to medicines in the March 2013 issue of Pharmaceutical Patent Analyst (Pharmaceutical Patent Analyst (2013) 2:2 (195-213)) | 2013 | Anonymous |
| 157 | European court opens a small window of opportunity for pharmaceutical companies to restrict parallel imports of medicines | 2004 | Hatton, C. and Nauwelaerts, W. |
| 158 | Expanding access and ensuring availability of affordable essential medicines for childhood cancer | 2012 | Auste, J. V. and Auste, C. V. |
| 159 | Financing free and universal access to antiretroviral drugs in the long-run: ART cost evolution in Brazil | 2013 | Meiners-Chabin, C. |
| 160 | Formulation changes will not stop parallel importing in Europe | 2002 | Anonymous |
| 161 | Generic medicines entry into the Malaysian pharmaceutical market | 2016 | Fatokun, O. and Ibrahim, M. I. M. and Hassaii, M. A. |
| 162 | Give the poor patients a chance: Enhancing access to essential medicines through compulsory licensing | 2008 | Kuanpoth, J. |
| 163 | Government use licenses in Thailand: The power of evidence, civil movement and political leadership | 2011 | Wibulpolprasert, S. and Chokevivat, V. and Oh, C. and Yamabhai, I. |
| 164 | Hepatitis C treatment on a shoestring | 2020 | Andrieux-Meyer, I. |
| 165 | HPR36 Barriers to Getting New Cancer Medicines to Patients in Kazakhstan | 2024 | Kostyuk, A. and Nurgozhin, T. and Almadiyeva, A. |
| 166 | Impact of Global Policy Reforms on Equity in Access to Medicines in India | 2016 | Gupta, S. |
| 167 | India adds compulsory drug licenses | 2013 | Anonymous |
| 168 | Indirect and direct savings resulted from parallel trade of pharmaceuticals in Poland - Results of valuation sales data from public pharmacies | 2013 | Baran-Lew and owska, I. and Hermanowski, T. |
| 169 | Indonesia government granted 7 compulsory licences to promote acces to HIV related medicines | 2012 | Velasquez, G. |
| 170 | Indonesian patent policy on compulsory license and access to affordable medicines | 2020 | Barizah, N. |
| 171 | Intellectual Property and Access to Medicines and Vaccines | 2022 | Velasquez, G. |
| 172 | Intellectual Property Rights and Vaccines | 2022 | Gilbert, P. and Fawcett, R. and Coles, J. and Hillson, W. |
| 173 | Introduction: AIDS and Africa | 2002 | Kopelman, L. M. and Van Niekerk, A. A. |
| 174 | Malaysia's Hepatitis C Elimination Journey - Global Implications and Opportunities | 2023 | Hassan, M. R. A. |
| 175 | Midterm Outcome Evaluation of Government-Led Endeavors to Eliminate Hepatitis C (HCV) as a Public Health Threat by 2030 in Malaysia | 2021 | Chan, H. K. and Hassali, M. A. and Md Said, R. and Omar, H. and Mutalib, N. A. A. and De Rozario, F. W. and Hassan, M. R. A. |
| 176 | Mozambique issues compulsory license for HIV antiretrovirals | 2004 | Anonymous |
| 177 | On the Possibility of Eradicating Hepatitis C in Russia | 2021 | Kochneva, G. V. and Kartashov, M. Y. and Krivosheina, E. I. and Kuznetsov, A. I. and Chub, E. V. and Sivolobova, G. F. and Netesov, S. V. |
| 178 | An overview of compulsory licensing in pharmaceuticals | 2016 | Aggarwal, P. and Rao, B. S. and Ravi Kumar, M. |
| 179 | Papers from the First International Conference on Innovation and Intellectual Property Rights | 2022 | Bell, M. and T and on, R. |
| 180 | Parallel import of drugs in Germany: An overview of the market segment of parallel imports and a forecast of its development. [German] | 2011 | Frenzel, A. and Maier, A. and Weissenfeldt, F. |
| 181 | Parallel importation as a policy option to reduce price of patented health technologies | 2021 | Abbas, M. Z. |
| 182 | Parallel imports save the NHS 228m | 2003 | Anonymous |
| 183 | Parallel imports: The growing market | 2017 | O'Kane, R. and Benton, V. |
| 184 | Parallel trade of medicinal products on the Croatian market. [Croatian] | 2021 | Cvek, J. and Mihalic, V. and Perojevic, Z. S. |
| 185 | Parallel trade of pharmeuticals in Poland | 2009 | Baran-Lew and owska, I. and Hermanowski, T. |
| 186 | Patenting of pharmaceuticals: An Indian perspective | 2012 | Mathur, V. |
| 187 | Patents, access to medicines and the role of non-governmental organisations | 2004 | Ford, N. |
| 188 | Pathways to ensure universal and affordable access to hepatitis C treatment | 2018 | Douglass, C. and Pedrana, A. and Lazarus, J. V. and T'Hoen, E. F. and Hammad, R. and Baptista-Leite, R. and Hill, A. and Hellard, M. |
| 189 | Pharma industry and patenting prosecution: An Indian perspective | 2018 | Khatri, S. and Walia, R. |
| 190 | Pharmaceutical compulsory licensing in emerging markets: Is it a welfare licensing or threat? | 2014 | Sharma, R. and Sahu, S. |
| 191 | Pharmaceutical patents and right to health. [Turkish] | 2019 | Ersoz Secer, H. and Sar, S. |
| 192 | Pharmaceutical Strategy for Europe: Emergency Use and Intellectual Property amid the COVID-19 pandemic | 2022 | Ussai, S. and Lauria, B. and Pistis, M. |
| 193 | PIN51 Out-of-Pocket Healthcare Expenditure in Various Household Income Groups Among Patients with Hepatitis C Disease Stages: Findings from a Tertiary Care Centre in Malaysia | 2020 | Azzeri, A. and Jaafar, H. |
| 194 | Pns70 Us "Import-from-Canada" Drug Legislation: Will It Lower Drug Costs for American Consumers? | 2020 | Maier-Downing, T. and Sabesan, P. and Jaszewski, B. |
| 195 | The potential impact of the Comprehensive and Progressive Agreement for Prans-Pacific Partnership on Thailand's hepatitis C treatment program | 2024 | Tenni, B. and Lexchin, J. and Akaleephan, C. and Kittitrakul, C. and Gleeson, D. |
| 196 | Pricing of drugs and donations: Options for sustainable equity pricing | 2001 | Perez-Casas, C. and Herranz, E. and Ford, N. |
| 197 | Raltegravir: Court of Justice confirms compulsory license | 2017 | Tebroke, E. |
| 198 | Recent developments in compulsory licensing of pharmaceutical Patents in India | 2016 | Konde, V. |
| 199 | Repackaging and Re-affixing of Pharmaceuticals in European Community Law. [Slovene] | 2003 | Bedrac, J. |
| 200 | Research on the policy orientation of China's drug patent compulsory license -based on a quantitative analysis for the implementation cases of global compulsory license of drug patent. [Chinese] | 2016 | Ding, J. X. and Yao, X. F. and Liu, W. J. |
| 201 | Review of the cases on the implementation of drug patent compulsory license from the international perspective. [Chinese] | 2020 | Cao, L. and Liu, Z. and Zhao, K. and Liu, Y. H. |
| 202 | The role of intellectual property rights in treatment access: Challenges and solutions | 2013 | E, 'T Hoen and Passarelli, C. A. |
| 203 | Sofosbuvir: Treatment of chronic hepatitis c and the main trends in patent protection | 2019 | Litvinova, E. V. and Posilkina Olga, V. and Maslova Nataliia, F. |
| 204 | Supplying pharmaceuticals to countries without manufacturing capacity: Examining the solution agreed upon by the WTO on 30th August, 2003 | 2004 | Correa, C. M. |
| 205 | The thai experience to overcome high cost drug in cancer | 2017 | Thongprasert, S. |
| 206 | Treatment coverage and drug expenditure in hepatitis c patients from 2013 to 2019: A journey of improving treatment accessibility in malaysia through government-led initiatives | 2020 | Chan, H. K. and Hassali, M. A. and Said, R. M. and Hassan, M. R. A. |
| 207 | TRIPS post-2005 and access to new antiretroviral treatments in southern countries: Issues and challenges | 2007 | Orsi, F. and D'Almeida, C. and Hasenclever, L. and Camara, M. and Tigre, P. and Coriat, B. |
| 208 | Using TRIPS-flexibilities as a leverage to improve access to HIV and hepatitis C medicines in Ukraine | 2018 | Kondratyuk, S. |
| 209 | Waiving COVID-19 vaccine patents: A bad idea and a dangerous precedent | 2021 | Pitts, P. J. and Popovian, R. and Winegarden, W. |
| 210 | What about parallel imports? | 2009 | Melnick, P. |
| 211 | Whither to public interest-the curious case of compulsory drug licensing in indian pharmaceutical industry | 2020 | Sehgal, R. K. and Koul, R. L. |
| 212 | World trade organization's export-oriented compulsory licensing mechanism: Foreseen policy concern for Africa to mitigate the COVID-19 pandemic | 2021 | Abbas, M. Z. |

### Annex S4: Details of Article 31bis Cases and Civil Society Instances

| Country | Year | Product | Executed | Explanation |
| --- | --- | --- | --- | --- |
| Canada (export) | 2004 | Imatinib | No | Applicant never received a response ([ref](https://www.keionline.org/wp-content/uploads/KEI-Briefing-Note-2021-2-CAMR-Canadian-Compulsory-Licensing.pdf)) |
| Canada (export) | 2006 | Oseltamivir | No | 7 month delay in amending Schedule 1 (ref) |
| Canada (export) | 2007 | 3TC/AZT/NVP | Yes | For export to Rwanda, who relied on LDC measure (paragraph 7) to fulfil the import responsibility (refs) |
| Bolivia* (import) | 2021 | COVID-19 vaccine | No | Depended on Canada to issue the CL for export (ref) |
| Canada* (export) | 2021 | COVID-19 vaccine | No | COVID-19 vaccines not added to Schedule 1 [[ref](https://www.keionline.org/wp-content/uploads/Request_to_Amend_Schedule_1_27April2021.pdf) [ref](https://laws-lois.justice.gc.ca/eng/acts/p-4/page-17.html#h-413972)] |

*All Known Article 31bis instances and Outcomes, 2001-2024*

** denotes paired cases (importing and exporting countries)*

*3TC/AZT/NVP = combination therapy of Lamivudine, Azidothymidine, Nevirapine for treatment of HIV/AIDS*

| Non-executed CLs: Outcomes, 2001-2024 | | | | | | | | | | | | | | | | | |
| --- | --- | --- | --- | --- | --- | --- | --- | --- | --- | --- | --- | --- | --- | --- | --- | --- | --- |
| **WTO Country Classification** | **Voluntary License** | | **Discount/ Donation** | | **Regulatory Barriers** | | **No Response** | | **Rejection** | | **Withdrawn** | | **Other** | | **Total** | | |
| **Column1** | **n** | **(%)** | 2n | (%)2 | 3n | (%)3 | 4n | (%)4 | 5n | **(%)**5 | 6n | **(%)**6 | 7n | **(%)**7 | 8n | **(%)**8 |  |
| **Total** | 8 | (16.3) | 15 | (30.6) | 4 | (8.2) | 8 | (16.3) | 10 | (20.4) | 2 | (4.1) | 2 | (4.1) | 49 | (100) |  |
| HIC | 2 | (8.7) | 6 | (26.1) | 3 | (13.0) | 5 | (21.7) | 5 | (21.7) | 1 | (4.3) | 1 | (4.3) | 23 | (46.9) |  |
| DC | 6 | (23.1) | 9 | (34.6) | 1 | (3.8) | 3 | (11.5) | 5 | (19.2) | 1 | (3.8) | 1 | (3.8) | 26 | (53.1) |  |
| LDC | 0 |  | 0 |  | 0 |  | 0 |  | 0 |  | 0 |  | 0 |  | 0 |  |  |
| Observer | 0 |  | 0 |  | 0 |  | 0 |  | 0 |  | 0 |  | 0 |  | 0 |  |  |

*Breakdown of Non-Executed CLs by WTO Country Classification and Outcome, 2001-2024*

| CL instances resulting from Civil Society campaigning: Outcomes, 2001-2024 | | | | | | | | | | | | | | | | |
| --- | --- | --- | --- | --- | --- | --- | --- | --- | --- | --- | --- | --- | --- | --- | --- | --- |
| **WTO Country Classification** | **Executed** | | **Pending** | | **Not executed** | | | | | | | | | | | |
|  |  |  |  |  | **Discount/ Donation** | | **Regulatory Barriers** | | **No Response** | | **Rejection** | | **Other** | | **Total** | |
| **Column1** | **n** | **(%)** | 2n | (%)2 | 3n | (%)3 | 4n | (%)4 | 5n | **(%)**5 | 6n | **(%)**6 | 8n | **(%)**8 | 9n | **(%)**9 |
| **Total** | 3 | (9.4) | 7 | (21.9) | 8 | (25.0) | 2 | (6.3) | 5 | (15.6) | 6 | (18.8) | 1 | (3.1) | 32 | (100.0) |
| HIC | 0 |  | 1 | (7.1) | 3 | (21.4) | 2 | (14.3) | 3 | (21.4) | 4 | (28.6) | 1 | (7.1) | 14 | (43.8) |
| DC | 3 | (16.7) | 6 | (33.3) | 5 | (27.8) | 0 |  | 2 | (11.1) | 2 | (11.1) | 0 |  | 18 | (56.3) |
| LDC | 0 |  | 0 |  | 0 |  | 0 |  | 0 |  | 0 |  | 0 |  | 0 |  |
| Observer | 0 |  | 0 |  | 0 |  | 0 |  | 0 |  | 0 |  | 0 |  | 0 |  |

*Breakdown of CLs instances resulting from Civil Society campaigning by outcome, 2001-2024*
