# Supplementary material for: TRIPS flexibilities help change policy and practice to increase access to medicines: evidence from 2001 to 2024
Source: BMJ Glob Health. 2026 Jan 28;11(1):e021481. doi: 10.1136/bmjgh-2025-021481 (PMC12853504; doi:10.1136/bmjgh-2025-021481)
Supplement: online supplemental file 2 [file bmjgh-11-1-s002.docx]

***TRIPS flexibilities help change policy and practice to increase access to medicines: Evidence from 2001-2024***

Montgomery Dunn, Ellen ‘t Hoen, Pascale Boulet, Kaitlin Mara, Katrina Perehudoff

## **Glossary**

## **Acronyms**

**CL** – Compulsory license/licensing

**DC** – Developing country

**HIC** – High-income country

**IP** – Intellectual property

**LDC** – Least-developed country

**LMIC** – Low and middle-income country

**ML&P** – Medicines Law & Policy

**MPP** – Medicines Patent Pool

**NCD** – Non-communicable disease

**PEPFAR** – (United States) President's Emergency Plan for AIDS Relief

**PI** – Parallel import(ation)

**TRIPS** – The WTO Agreement on Trade-Related Aspects of Intellectual Property Rights

**UMIC** – Upper middle-income country

**USAID** – United States Agency for International Development

**WHO** – World Health Organization

**WTO** – World Trade Organization

## **Legal measures**

See Annex

## **Classifications**

### **WTO country classifications**

### The WTO maintains a list of LDCs. Countries self-identify as ‘developing’ within the framework of the WTO. This study classified all other countries as HICs. The WTO maintains a list of Members and Observers.

### **Instance outcome status**

**Executed** – The flexibility was enacted, regardless of the access outcome or whether supply was established. I.e. a compulsory license was issued or the LDC pharmaceutical transition waiver was invoked.

**Non-executed** – The flexibility was not used. I.e. a compulsory license was formally requested but not granted.

**Voluntary license** – The patent holder grants a third party the right to make use of the IP under certain conditions.

**Discount/donation** – The patent holder agrees to sell the product at a reduced price or donate a quantity of product.

**Regulatory barriers** – An array of issues caused by regulatory hurdles, such as data exclusivity provisions in medicines law.

**No response** – The government authority did not respond to the formal request for the use of a TRIPS flexibility, or no such response can be found. Instances are labelled as ‘no response’ if they have been pending for over 5 years.

**Rejection** – The government authority rejected the application for flexibility use.

**Withdrawn** – The applicant withdrew the request for flexibility use.

**Other** – Any outcome that does not fit into the above categories, e.g. there was no applicable patent in the territory.

**Pending** – The instance is less than 5 years old and none of the above outcomes (except ‘no response’) yet apply.

### **Instance characteristics**

**Applicant/licensee** – Either: the non-governmental party that submitted a formal request for the use of a flexibility or, in cases where there was no formal request from such a party, the government itself.

**Civil Society** – An organization or individual not acting on behalf of government or business.

**Government** – The government of the applicable country.

**Pharmaceutical Company** – A business that develops, produces and markets pharmaceutical products.

**Procurement agent** – A business, not-for-profit organisation or UN Agency that sources and supplies pharmaceutical products but does not produce them themselves.

**Unknown** – The applicant/licensee is not mentioned in the available information regarding the instance.
